# Mutations in Na_V_1.5 Reveal Calcium-Calmodulin Regulation of Sodium Channel

**DOI:** 10.3389/fphys.2019.00700

**Published:** 2019-06-05

**Authors:** Eyal Nof, Leonid Vysochek, Eshcar Meisel, Elena Burashnikov, Charles Antzelevitch, Jerome Clatot, Roy Beinart, David Luria, Michael Glikson, Shimrit Oz

**Affiliations:** ^1^Heart Center, Sheba Medical Center, Ramat Gan, Israel; ^2^Sackler School of Medicine, Tel Aviv University, Tel Aviv, Israel; ^3^Lankenau Institute for Medical Research, Wynnewood, PA, United States; ^4^Lankenau Heart Institute, Wynnewood, PA, United States; ^5^Sidney Kimmel Medical College, Thomas Jefferson University, Philadelphia, PA, United States

**Keywords:** β1-subunit, calmodulin, cardiac arrhythmia, channelopathies, DIII-IV linker, heart, *SCN5A*, sodium current

## Abstract

Mutations in the *SCN5A* gene, encoding the cardiac voltage-gated sodium channel Na_V_1.5, are associated with inherited cardiac arrhythmia and conduction disease. Ca^2+^-dependent mechanisms and the involvement of β-subunit (Na_V_β) in Na_V_1.5 regulation are not fully understood. A patient with severe sinus-bradycardia and cardiac conduction-disease was genetically evaluated and compound heterozygosity in the *SCN5A* gene was found. Mutations were identified in the cytoplasmic DIII-IV linker (K1493del) and the C-terminus (A1924T) of Na_V_1.5, both are putative CaM-binding domains. These mutants were functionally studied in human embryonic kidney (HEK) cells and HL-1 cells using whole-cell patch clamp technique. Calmodulin (CaM) interaction and cell-surface expression of heterologously expressed Na_V_1.5 mutants were studied by pull-down and biotinylation assays. The mutation K1493del rendered Na_V_1.5 non-conductive. Na_V_1.5_K1493del_ altered the gating properties of co-expressed functional Na_V_1.5, in a Ca^2+^ and Na_V_β1-dependent manner. Na_V_1.5_A1924T_ impaired Na_V_β1-dependent gating regulation. Ca^2+^-dependent CaM-interaction with Na_V_1.5 was blunted in Na_V_1.5_K1493del_. Electrical charge substitution at position 1493 did not affect CaM-interaction and channel functionality. Arrhythmia and conduction-disease -associated mutations revealed Ca^2+^-dependent gating regulation of Na_V_1.5 channels. Our results highlight the role of Na_V_1.5 DIII-IV linker in the CaM-binding complex and channel function, and suggest that the Ca^2+^-sensing machinery of Na_V_1.5 involves Na_V_β1.

## Introduction

Sodium current (I_Na_) upstroke is a hallmark of the action-potential in excitable cells. The *SCN5A* gene encodes the pore-forming α-subunit of the cardiac sodium channel Na_V_1.5. Na_V_1.5 channels are expressed in cardiomyocytes as well as the cardiac His-Purkinje system. Accordingly, Na_V_1.5 loss-of-function mutations are associated with cardiac arrhythmia and conduction defects (Schott et al., 1999; Tan et al., 2001; Holst et al., 2010; Zumhagen et al., 2013). Although I_Na_ does not contribute to the action potential of pacemaker cells, its presence in the periphery of the sinoatrial node can modulate impulse conduction and heart rate (Kodama et al., 1997). Hence, loss-of-function mutations in *SCN5A* can result in a nodal dysfunction (Benson et al., 2003; Lei et al., 2008; Gui et al., 2010; Ziyadeh-Isleem et al., 2014; Chiang et al., 2015; Milanesi et al., 2015).

**FIGURE 1:**
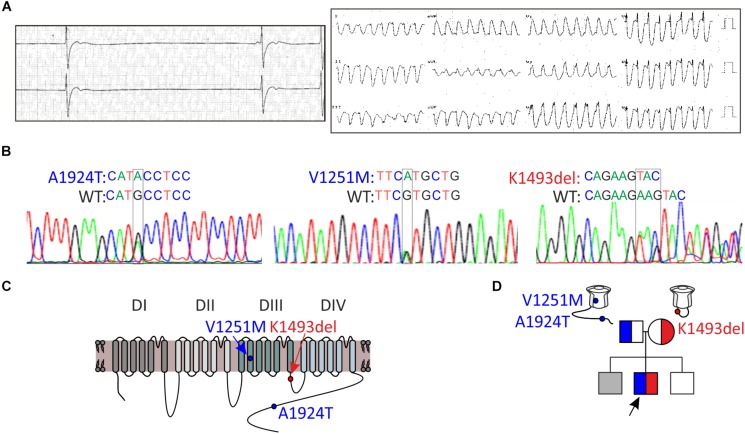
Clinical and genetic characteristics of the proband. **(A)** Left, Holter recording at rest. Right, 12-lead ECG during exercise. **(B)** Sequence chromatogram of *SCN5A* variants. **(C)** Schematic illustration of Na_V_1.5 protein and the location of the variants found. **(D)** Pedigree tree of the family. Circle indicates female, squares indicate male. The arrow points to the proband. In gray, suspected affected sibling. Red for the maternal allele variant, blue for the paternal allele variant.

The Na_V_1.5 α-subunit is composed of four homologous domains (DI-DIV), each containing six transmembrane repeats. The domains are joined by three cytosolic linkers and flanked by cytosolic C- and N-termini (see Figure 1C). The α-subunit forms a functional monomer; however, multi-channel assembly and functional coupling among monomeric channels have been reported (Keller et al., 2005; Poelzing et al., 2006; Clatot et al., 2017). Moreover, the α-subunit is the core of a macromolecular complex that interacts with auxiliary proteins that modulate expression, trafficking, localization and gating of Na_V_1.5; among them the regulatory β-subunits (Na_V_β) and the Ca^2+^-sensor calmodulin (CaM) (Abriel, 2010).

Na_V_1.5 is regulated by Ca^2+^, but the role and mechanism of this regulation is still debated (Van Petegem et al., 2012; Ben-Johny et al., 2014; Gabelli et al., 2016; Pitt and Lee, 2016; Johnson, 2019). CaM interacts with Na_V_1.5 C-terminus (CT) (Gabelli et al., 2014; Wang et al., 2014), and the linker between domains DIII-IV (DIII-IV linker) (Sarhan et al., 2012; Johnson et al., 2018). However, the contribution of each CaM-interaction domain to the Ca^2+^-sensing machinery of Na_V_1.5 is not fully established. Na_V_β1 interacts non-covalently with Na_V_1.5 and can modulate I_Na_ gating, as well as transcription and cell-adhesion (Calhoun and Isom, 2014). The involvement of Na_V_β1 in Ca^2+^-dependent gating regulation is not clear.

We report a novel combination of *SCN5A* variants, K1493del and A1924T, in a patient with sinus-bradycardia and cardiac conduction-disease. Heterozygous A1924T has been previously associated with Brugada syndrome (BrS) (Rook et al., 1999), and homozygous A1924T with sinus-bradycardia with conduction delay (Lopez et al., 2011). Heterozygous K1493del has been associated with isolated conduction disease (Zumhagen et al., 2013). Here, we studied the molecular basis of Ca^2+^- and CaM-dependent Na_V_1.5 modulation, using the disease-associated mutations.

## Materials and Methods

### Clinical

The proband and his parents gave written informed consents for both the clinical and genetic studies, which were approved by the Institutional Ethics-Committee of the Sheba Medical Center, Tel-Hashomer (approval 2853/03). Evaluation included resting electrocardiogram (ECG), 24-h Holter monitoring (DELMAR^®^ systems; Impresario 3.04.0089), two-dimensional echocardiography, treadmill exercise test, cardiac MRI, ajmaline test and an electrophysiological-study.

### Genetic Analysis

Heparinized blood was drawn from the proband and his parents; DNA was extracted using a commercial kit (Gentra System Inc., Minneapolis, MN, United States) and the exons and exon-intron boundaries of the following genes: *HCN4*, *KCNJ2*, *KCNJ12*, and *SCN5A* were amplified by PCR (Verities PCR, Applied Biosystems, Austin, TX, United States). The PCR products were purified (Exosap-IT, USB, Isogen Life-Science, Netherlands) and sequenced in both directions (BigDye Terminator v3.1 cycle sequencing Kit and 3130xL Genetic Analyzer, Applied Biosystems).

### Molecular Biology

A DNA construct of the paternal Na_V_1.5 variant was prepared with two variants, A1924T and V1251M, on the same DNA construct and denoted as Na_V_1.5_A1924T^*_. In the maternal channel, Na_V_1.5_K1493del_, one lysine of the doublet in positions 1492-3 was deleted. N-terminally tagged green-fluorescent protein (GFP)-Na_V_1.5 was used (Zimmer et al., 2002). In the constructs K1493A/E/R the second lysine of the lysine-doublet was substituted with the amino-acids indicated. Point-mutations were prepared by site-directed mutagenesis using a standard PCR (Roche, IN, United States), and followed by sequencing of the entire coding sequence. Rat and human -Na_V_β1 (rNa_V_β1 and hNa_V_β1, respectively) were cloned into pcDNA3 vector using HindIII and EcoRI sites, and followed by an internal ribosome entry site (IRES) and the red fluorescent-protein (RFP) sequence between EcoRI and NotI sites (rNa_V_β1/RFP and hNa_V_β1/RFP, respectively). Na_V_1.5 (NM_198056.2), calmodulin (M19312), rNa_V_β1 (M91808) hNa_V_β1 (NP_001028), and GFP were all in a pcDNA3 vector.

### Cell Culture

**FIGURE 2:**
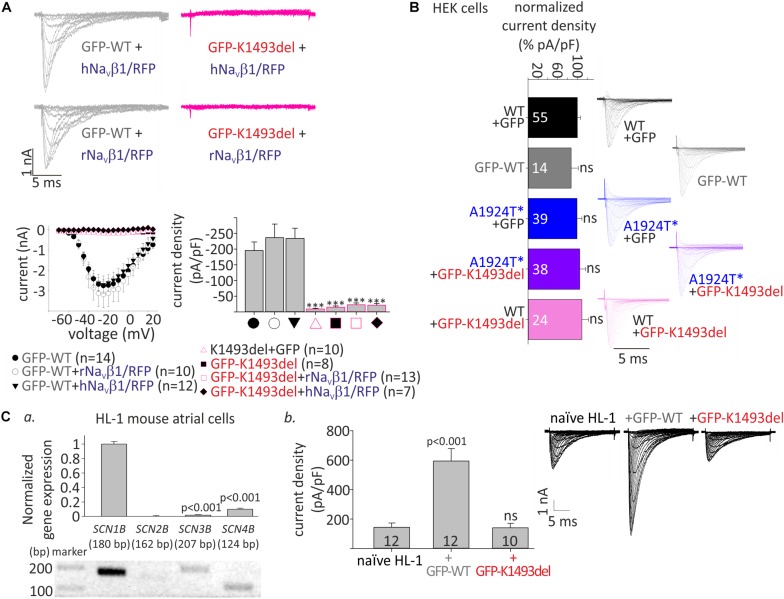
Whole-cell I_Na_ of WT and mutant Na_V_1.5 in HEK and HL-1 cells. **(A)** Top, representative currents elicited from resting potential by depolarizing steps (–70 until –20 mV in 5 mV interval) in HEK cells. Bottom, voltage-dependent amplitudes and maximal current densities, in cells expressing GFP-Na_V_1.5_WT_, GFP-Na_V_1.5_K1493del_, and Na_V_1.5_K1493del_, with and without r/hNa_V_β1 co-expressing RFP in the same vector. All groups were compared to GFP-WT for statistical significance. **(B)** WT and mutant Na_V_1.5 expressed in HEK cells (without Na_V_β subunit). Current densities were normalized to the mean current density of Na_V_1.5_WT_ in the same experiment and summarized from 9 experiments. Currents in A and the representative currents in B were recorded in [10 mM EGTA]_in_. **(Ca)**, Gene expression levels of *SCNB1-4* in HL-1 cells. Top, gene levels were quantified by real-time PCR and normalized to GAPDH. Bottom, semi-quantitative PCR. **(b)**, I_Na_ measured in naïve and transfected HL-1 cells, in [10 mM EGTA]_in_. Mean current densities and representative cells are shown. Statistical significance was determined by one-way ANOVA, followed by Holm–Sidak test. ns, not significant.

HEK293 cells were maintained in Dulbecco’s Modified Eagle’s Medium supplemented with 10% Fetal Bovine Serum, 100 U/ml penicillin, 10 mg/ml streptomycin and 2 mM L-Glutamine (Biological Industries, Kibbutz Beit-Haemek, Israel) at 37°C with 5% CO_2_. For electrophysiological experiments, transfections were performed in 35 mm dish using *Trans-*ITx2 (Mirus, Madison, WI, United States) according to the manufacturer’s instructions. 1 μg of each construct was used for transfection (Na_V_1.5, Na_V_β1/RFP, and CaM), except in Figure 2A, where 3 μg of GFP tagged- K1493del mutated -Na_V_1.5 (GFP-Na_V_1.5_K1493del_) and Na_V_β1/RFP (μg DNA ratio 1:1) were used. When indicated, 0.5 μg GFP was added as a transfection marker. On the following day, cells were plated on coverslips. For biochemical experiments, cells in 10-cm dishes were transfected using the Calcium-Phosphate method. 5–15 μg DNA of Na_V_1.5 α-subunit were used, rNa_V_β1 was added in a 0.6 β:α molar ratio unless otherwise indicated. In Figure 5B, CaM was added in a 2 CaM:α molar ratio. Experiments were performed 48–72 h after transfection.

HL-1 cells were plated in gelatin/fibronectin-coated dishes, in Claycomb media supplemented with 10% Fetal Bovine Serum, 100 μM norepinephrine (Sigma, St. Louis, Mo, United States), 100 U/ml penicillin, 10 mg/ml streptomycin and 2 mM L-Glutamine (Biological Industries). Transfections using Lipofectamine 3000 (Invitrogen, Carlsbad, CA, United States) were performed in 35 mm dishes. On the following day, cells were plated on gelatin/fibronectin-coated coverslips. Experiments were performed 48 h after transfection.

### RNA Extraction and Quantitative PCR

Total RNA was extracted using RNeasy Plus mini kit (Qiagen, Hilden, Germany). Reverse transcription with random primers was performed using the high capacity cDNA reverse transcription kit (Applied Biosystems, CA, United States). cDNA was amplified using KAPA HiFi HotStart ReadyMix (Roche). Quantitative real-time PCR was performed using ABI Step-one plus sequence detection system (Applied Biosystems) with Fast SYBR Green Master Mix reagent (Applied Biosystems). The primers for mouse genes used were, for Na_V_β1 (NM_011322.2): SCN1B Fw (CGAGGCTGTGTATGGGATGAC)/SCN1B Rv (CCCTCAAAGCGCTCATCTTC), for Na_V_β2 (NM_001014761.2): SCN2B Fw (GTGAACCACAAGCAGT TCTCT)/SCN2B Rv (TGACACGTCGTACTTACTGGG), for Na_V_β3 (NM_001286614.1): SCN3B Fw (TGTAATGTG TCCAGGGAGTTTG)/SCN3B Rv (TTCGGCCTTAGAGACCT TTCTG) and for Na_V_β4 (NM_001013390.3): SCN4B Fw (GGAACCGAGGCAATACTCAGG)/SCN4B Rv (CCGTTAA TAGCGTAGATGGTGGT). Gene expression was quantified using the 2^−ΔΔCt method by normalization to the housekeeping gene GAPDH (Fw: TCGTCCCGTAG ACAAAATGG/Rv: TTGAGGTCAATGAAGGGGTC).

### Electrophysiology

Currents were recorded using the whole-cell configuration of the patch-clamp technique at 23°C. Red- and/or green- fluorescent cells were selected for recording. Signals were amplified using an Axopatch 200B patch-clamp amplifier (Axon Instruments, Foster City, CA, United States), sampled at 100 kHz and filtered with a low-pass Bessel filter at 10 kHz. Data was acquired with DigiData1440A and analyzed using pCLAMP 10 software (Axon Instruments). Patch pipettes (Harvard apparatus) resistance was 1.5–4 MΩ. Cells with access resistance over 5 MΩ were discarded. Series resistance was compensated by 85%. Leak currents were subtracted using a P/3 protocol. External solution contained (in mM) 137 NaCl, 4 KCl, 10 Hepes, 10 Glucose, 1.8 CaCl_2_, and 1 MgCl_2_ titrated with NaOH to pH 7.4, and adjusted to 310 mOsm. Internal solution contained (in mM) 100 CsF, 25 NaF, 10 Hepes, 10 NaCl, 2 Mg^2+^-ATP and 10 ethylene glycol-bis (β-aminoethyl ether)-N,N,N′,N′-tetraacetic acid (EGTA), titrated with CsOH to pH 7.2 and adjusted to 295 mOsm, this pipette solution is (10 mM EGTA)_in_. For fast Ca^2+^ chelation, EGTA was replaced with 10 mM 1,2-BIS (2-AMINOPHENOXY)-ETHANE-N,N,N′N′-TETRAACETIC ACID [(10 mM BAPTA)_in_]. For pipette solution with 10 μM Ca^2+^, EGTA was replaced with 1 mM BAPTA and 1 mM CaCl_2_ [(10 μM Ca^2+^)_in_]. EGTA was replaced with 5 mM N-(2-Hydroxyethyl)ethylenediamine-N,N′,N′-triacetic acid (HEDTA) and 2.5 mM CaCl_2_ for [10 μM Ca^2+^]_in_/HEDTA (calculated with WebMaxC extended program^1^). Pronase (Roche) was dissolved in [10 mM EGTA]_in_ solution.

Activation protocol was initiated after 2 min, steady-state inactivation (SSI) protocol after 3.25 min and recovery from inactivation after 8 min from the rupture of the cell membrane.

Holding potential was −120 mV. Current-densities were obtained by dividing the peak current by the cell capacitance. Normalized current-densities were calculated by dividing the current density of each cell with the mean Na_V_1.5_WT_ current density measured on the same day of the experiment, using the same pipette solution composition. The voltage-dependent activation was calculated by fitting currents, generated by steps from −80 to 40 mV in 5 mV increments for 20 ms, with a modified Boltzmann equation: I = [Gmax^*^(V-Vrev)]/[1+exp(Va-V)/k], where I is the peak current for the test potential V, Gmax is maximum conductance, Vrev is the reversal potential, Va is the potential for half-activation or half-availability, and k is the slope factor. The normalized conductance was determined by modified Ohm’s law G/Gmax = I/Gmax (V-Vrev).

Steady-state inactivation was assayed by a 20 ms test pulse to −20 mV after a 500 ms pre-pulse to varying voltages (from −140 to −45 mV in 5 mV steps). SSI curves were fitted with Boltzmann equation: I = 1/[1 + exp(Va − V)/k],

Recovery from inactivation curve was obtained by a 1 s conditioning pulse (I1) to -20 mV followed by a test pulse (I2) to -20 mV after a varying time (1–24 ms, with 1 ms steps) at -120 mV. Fractional recovery was calculated as I2/I1. The time constant (τ) and amplitude (A) of recovery from inactivation were obtained by fitting the data with the function y = A(1−e^–t/τ^).

Waveform’s current decay was fitted to one -exponent fit using Levenberg-Marquardt algorithm, in the form f(t) = Ae^–t/τ^ +C.

### Cell Surface Biotinylation and CaM-Beads Pull-Down

For biotinylation experiments, cells were incubated with 0.5 mg/ml EZ-Link Sulfo-NHS-SS-Biotin (Pierce, Rockford, IL, United States) in phosphate buffer saline (pH 8) containing 1 mM Ca^2+^ and 0.5 mM Mg^2+^ (PBS-CM), for 30 min at 4°C. The reaction was terminated by incubation in 50 mM glycine in PBS-CM, for 10 min at 4°C. Cells were scraped and washed with PBS-CM supplemented with 0.5 mM phenylmethylsulfonyl fluoride (PMSF) at 4°C. Cells were lysed in TNE buffer (in mM): 20 Tris–HCl pH 8, 150 NaCl, 1 EGTA, and 1% NP40 supplemented with 0.5 PMSF, protease inhibitor mixture (Roche), 25 β-glycerol phosphate and 1 Na_3_VO_4_, for 30 min on ice. A 25 μg sample was taken for input. An equal amount of total protein from each group was incubated with streptavidin-agarose beads (Pierce) for 2 h at 4°C in a rotating device. Samples were washed with TNE buffer supplemented with 0.5 mM PMSF, and proteins were eluted by incubation with sample buffer and freshly added 100 mM DTT, for 1 h at room temperature, and then for 5 min at 65°C. Na_V_1.5_WT_ and Na_V_1.5_K__1493del_ intensities were compared in groups transfected with an equal amount of DNA.

For CaM pull-down experiments, cells were collected in PBS supplemented with 0.5 mM PMSF, and lysed with (in mM) 20 Tris–HCl pH 7.5, 150 NaCl, and 1% Triton x100, supplemented with 0.5 PMSF, protease inhibitor mixture (Roche), 25 β-glycerol phosphate and 1 Na_3_VO_4_, for 30 min on ice. A 25 μg sample was taken for input. CaM agarose beads (A6112, Sigma) were washed with (in mM) 20 Tris–HCl pH 7.5, 150 NaCl added with either 2 CaCl_2_, or 10 EGTA, then were incubated with an equal amount of total protein from each group, supplemented with either 2 CaCl_2_ or 10 EGTA, for 2 h at 4°C in a rotating device. Samples were washed with (in mM) 20 Tris–HCl pH 7.5, 150 NaCl and 1% Triton x100 supplemented with 0.5 PMSF, and proteins were eluted with sample buffer, for 5 min at 65°C.

Normalized Na_V_1.5 intensity was calculated by dividing Na_V_1.5 intensity by a normalizer, γ-tubulin for inputs (Na_V_1.5-input_NORM_ = Na_V_1.5/γ-tubulin) and Na-K ATPase for plasma membrane-bound fraction (Na_V_1.5-PM_NORM_ = Na_V_1.5/Na-K ATPase). Percent of total expression was calculated by dividing intensities of Na_V_1.5-input_NORM_ with a control group from the same experiment. Trafficking was calculated by dividing Na_V_1.5-PM_NORM_ by Na_V_1.5-input_NORM_, and presented as % trafficking from the control group in the same experiment. CaM-beads binding level was quantified by dividing bound by input fractions and normalized to control group in the same experiment.

The antibodies that were used are hNa_V_1.5 (ASC-013, Alomone, Jerusalem, Israel), Na-K ATPase (ANP-001, Alomone), γ-tubulin (T5192, Sigma), and CaM (05-173, Millipore Corp., Temecula, CA, United States).

### Presentation and Statistical Analysis

Densitometry of Western blot bands was analyzed using ImageJ (NIH, United States). Figures were prepared using CorelDrawX8 (Corel Corp., Ottawa, Canada). Statistical analyses were performed using SigmaPlot 13 (Systat Software, CA, United States). Data are presented as mean ± SEM. One-way ANOVA followed by the multiple comparison Holm–Sidak *post hoc* test was used to compare several groups. Two-tailed Student’s *t*-test was used to compare two groups.

## Results

### Clinical and Genetic Data

A 16-year-old male presented with a syncope during exercise. Physical examination revealed a healthy looking young man without any abnormalities. He had bradycardia for many years but was not previously symptomatic. A 24-h Holter recording showed an average heart rate of 50 (range: 24–110) due to sinus-bradycardia and occasional junctional rhythm with sinus-pauses up to 5.8 s (Figure 1A, left). A wide complex tachycardia (WCT) of 193 beat-per-minute was recorded during exercise testing (Figure 1A, right). Echocardiography and cardiac-MRI did not reveal any structural abnormalities or areas of late gadolinium enhancement. Ajmaline provocation test ruled out BrS. Electrophysiological-study demonstrated a prolonged A-H interval of 260 ms and H–V interval of 75 ms. During rapid pacing (420 CL), the H–V interval increased to over 100 ms. Atrial-flutter was inducible with 1:1 conduction, leading to “clinical” WCT. The proband developed symptomatic bradycardia and a permanent pacemaker was eventually implanted.

DNA screening of the proband revealed compound heterozygosity in the *SCN5A* gene. The paternal allele had two missense variants, leading to amino-acid substitutions of alanine by threonine in position 1924 (A1924T) and valine by methionine in position 1251 (V1251M). The variant V1251M is a benign polymorphism (Kapplinger et al., 2015). The maternal allele had an in-frame, single codon deletion, resulting in a removal of one out of two adjacent lysines, in positions 1492-1493 (K1493del) (Figures 1B,C). We cannot rule out the presence of mutation(s) in additional gene(s) that were not identified in the present gene screen.

Holter-testing of both parents did not reveal bradycardia, but the father displayed premature ventricular contractions (PVCs) with Right Bundle Branch Block (RBBB) pattern in V1 on 12-lead ECG. One brother did not have bradycardia. Another brother had bradycardia but he declined any clinical or genetic evaluation (Figure 1D).

Although K1493del and A1924T heterozygotes were previously reported with bradyarrhythmias (Rook et al., 1999; Zumhagen et al., 2013), the heterozygotes in this report were asymptomatic, probably due to reduced disease penetrance in these individuals. The extent and reasons for *SCN5A* mutation expression variability are not entirely clear. Reasons for the variability may include epigenetic gene silencing, age, gender, environmental factors and other genetic modifiers (Liu et al., 2016; Verkerk et al., 2018). We suggest that the accumulated effects in the compound-heterozygote may have increased the penetrance and severity of the arrhythmic phenotype compared with the heterozygotes in the reported family.

### Complete Loss of Na_V_1.5 Activity Due to K1493del Mutation, Without a Dominant-Negative Effect on Current Density

To examine the functional consequences of the variants, we expressed Na_V_1.5, WT and mutants, in HEK cells and measured whole-cell I_Na_. We used N-terminally GFP-fused Na_V_1.5 that conserves the biophysical properties of Na_V_1.5 (Clatot et al., 2012; Reinhard et al., 2013). Peak I_Na_ recorded in cells transfected with GFP-Na_V_1.5_WT_, with or without Na_V_β1, was 1–6 nA. No I_Na_ was recorded in cells expressing GFP-Na_V_1.5_K1493del_ or the non-tagged Na_V_1.5_K1493del_. Addition of a bicistronic vector expressing Na_V_β1 from two species (rat or human) together with RFP as an expression reporter did not recover I_Na_ (Figure 2A), even when the amount of DNA used for transfection of GFP-Na_V_1.5_K1493del_ +Na_V_β1 was three-fold higher than GFP-Na_V_1.5_WT_. These results are at odds with a previous report (Zumhagen et al., 2013).

Compared with Na_V_1.5_WT_, the paternal variation Na_V_1.5_A1924T__^*_ did not affect I_Na_ density. In order to test whether Na_V_1.5_K1493del_ has a dominant-negative effect, GFP-Na_V_1.5_K1493del_ was added on top of Na_V_1.5_WT_ or Na_V_1.5_A1924T__^*_, in 1:1 DNA ratio. Co-expression of GFP-Na_V_1.5_K1493del_ did not reduce I_Na_ density, in HEK cells (Figure 2B and Table 1).

**TABLE 1:** Current densities (pA/pF) of the normalized values presented in Figure 2B.

WT + GFP (*n* = 55)	−235 ± 22
GFP-WT (*n* = 14)	−220 ± 24
A1924T^*^ + GFP (*n* = 39)	−247 ± 24
A1924T^*^ + GFP-K1493del (*n* = 38)	−211 ± 18
WT + GFP-K1493del (*n* = 24)	−160 ± 16

We used HL-1 cells, a mouse atrial cardiomyocyte tumor cell-line (Claycomb et al., 1998), to test the effect of Na_V_1.5_K1493del_ on endogenous cardiac-cell I_Na_. HL-1 cells express sodium channel subunits, predominantly the Na_V_1.5 α-subunit (Cerrone et al., 2014) and Na_V_β1 subunit (Figure 2Ca), that recapitulate the physiological Na_V_1.5 stoichiometry in a cardiac cell milieu. We transfected HL-1 cells with GFP-Na_V_1.5_WT_ or GFP-Na_V_1.5_K1493del_. Naïve, non-transfected, HL-1 cells showed I_Na_ of -144 ± 29 pA/pF. HL-1 cells transfected with GFP-Na_V_1.5_WT_ had I_Na_ of −594 ± 84 pA/pF, while cells transfected with GFP-Na_V_1.5 _K1493del_ had a basal I_Na_ of −140 ± 32 pA/pF, similar to non-transfected cells.

These findings demonstrate, in HEK and HL-1 cells, that Na_V_1.5_K1493del_ is a loss-of-function mutant that does not induce a functional dominant-negative effect on I_Na_ density.

### K1493del Attenuates Na_V_1.5 Expression but Does Not Affect Trafficking

**FIGURE 3:**
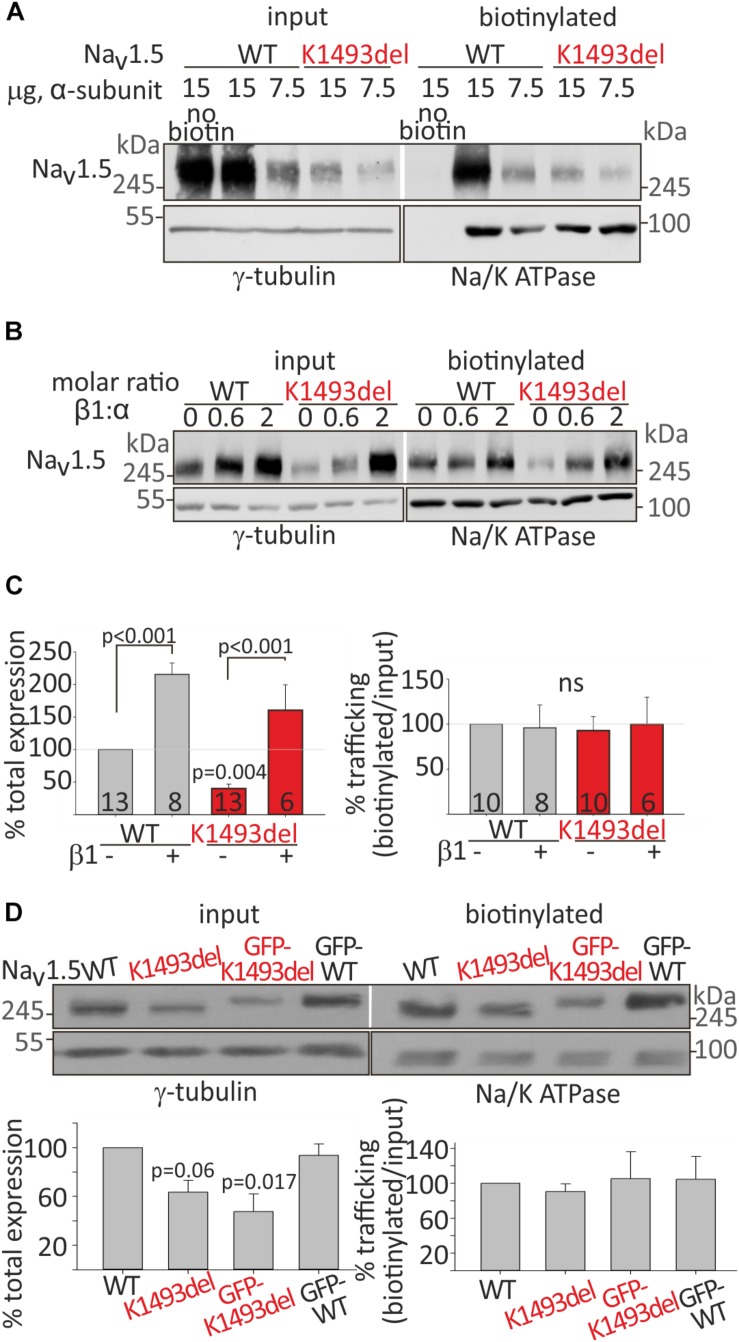
Cell surface and total expression of Na_V_1.5_WT_ and Na_V_1.5_K1493del_. **(A,B)** Representative biotinylation assays. **(A)** HEK cells were transfected with Na_V_1.5_WT_ and Na_V_1.5_K1493del_. The control “no-biotin” group shows no signal from non-biotinylated channels. **(B)** HEK cells transfected with Na_V_1.5_WT_ and Na_V_1.5_K1493del_ (7.5 μg) +/– rNa_V_β1 (2.5 and 7.5 μg). **(C)** Summary of % total protein (left) and % trafficking (right), normalized to Na_V_1.5_WT_ without Na_V_β1. *N* = 9 experiments without Na_V_β. *N* = 4 experiments with rNa_V_β1 expressed. Groups expressing rNa_V_β1 in molar ratios β:α 0.6-2 were pulled together. **(D)** GFP-fused and non-tagged Na_V_1.5_WT_ and Na_V_1.5_K1493del_ were expressed (without Na_V_β subunit). This is a summary of three experiments. Statistical significance was determined by one-way ANOVA, followed by Holm–Sidak test.

In order to understand if the basis for the loss-of-function in Na_V_1.5_K__1493del_ was an impairment in the protein expression or trafficking to the plasma membrane, we performed a biotinylation assay. Na_V_1.5_WT_ and Na_V_1.5_K__1493del_ were transfected in HEK cells. Total and plasma-membrane biotin-bound Na_V_1.5 protein levels were quantified following Western-blot. We found a reduction in total cellular expression of Na_V_1.5_K__1493del_ (40 ± 7% of Na_V_1.5_WT_). The % trafficking was determined by dividing the biotinylated fraction by total fraction, normalized to % trafficking of Na_V_1.5_WT_, in the same experiment. The % trafficking of Na_V_1.5_K1493del_ was not significantly different from % trafficking of Na_V_1.5_WT_ (93 ± 16%) so that a similar fraction from Na_V_1.5 total protein was present at the plasma membrane (Figures 3A,C), suggesting that trafficking of Na_V_1.5_K1493del_ to the plasma membrane was not impaired.

Na_V_β1 has been reported to improve the expression and trafficking of loss-of-function mutants of the sodium channel (Bechi et al., 2015). We examined the effect of Na_V_β1 co-expression on total and cell-surface expression of Na_V_1.5_WT_ and Na_V_1.5_K1493del_. Addition of Na_V_β1 enhanced total expression of both Na_V_1.5_WT_ and Na_V_1.5_K1493del_, with a concomitant increase in channel expression at the cell surface (Figure 3B).

A summary of total and biotinylated Na_V_1.5 levels, normalized to the Na_V_1.5_WT_ expressed without Na_V_β1 in the same experiment, is presented in Figure 3C. Co-expression of Na_V_β1 increased total expression of Na_V_1.5_WT_ by 215 ± 18% and Na_V_1.5_K1493del_ by ∼400%, increasing the latter from 40 ± 7% to 160 ± 39% (compared to Na_V_1.5_WT_ without Na_V_β1). Na_V_1.5_K1493del_ trafficking to the plasma membrane was similar to Na_V_1.5_WT_ when Na_V_β1 was co-expressed. These results support the conclusion that K1493del does not affect forward-trafficking or Na_V_β1-regulated trafficking and expression (Figure 3A).

To test whether the addition of a GFP-tag to Na_V_1.5 N-terminus affected the expression or trafficking of Na_V_1.5, we performed a biotinylation experiment on GFP-tagged and non-tagged channels. GFP-fusion did not change the biogenesis properties: total expression of GFP-Na_V_1.5_K1493del_ was partially reduced compared to GFP-Na_V_1.5_WT_, and GFP-tagged Na_V_1.5 channels were exported to the plasma membrane (Figure 3D).

In summary, K1493del partially reduced total protein expression but did not affect the plasma-membrane trafficking of Na_V_1.5. Co-expression of Na_V_β1 restored the reduced Na_V_1.5_K1493del_ cellular levels but did not restore I_Na_ (Figure 2A), thus the loss-of-function by K1493del mutation is only marginally due to a biogenesis defect.

### Expression of Non-conducting Na_V_1.5_K1493del_ and Na_V_β1 Affects Ca^2+^-Dependent Gating

**FIGURE 4:**
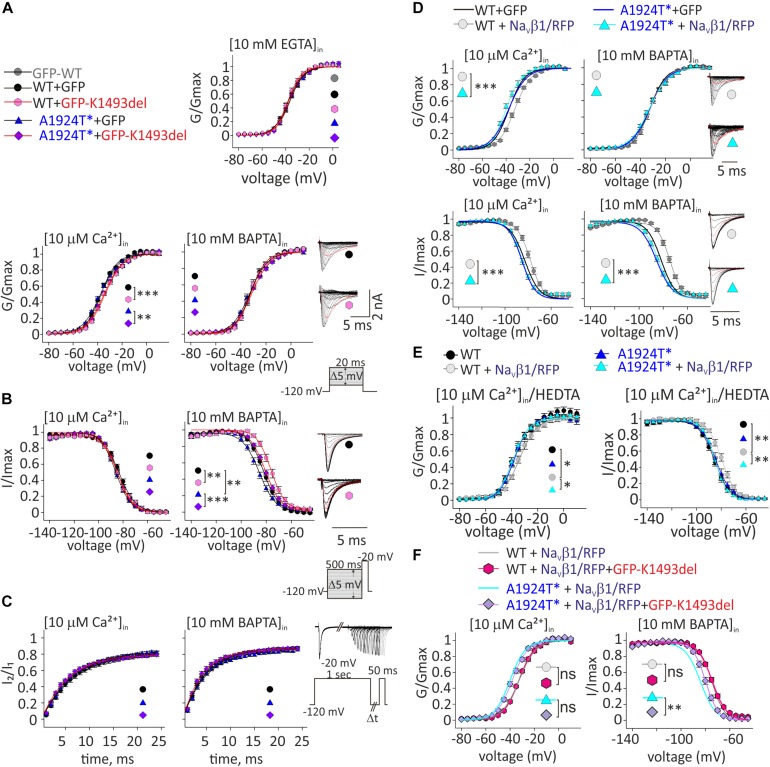
Ca^2+^-dependent gating properties of Na_V_1.5 variants. **(A)** Activation curves of conductance-voltage relationship. Current traces recorded in [10 μM Ca^2+^]_in_, and –40 mV trace is red. **(B)** SSI curves of voltage-dependent channel availability. Current traces during the test pulse at –20 mV are with (10 mM BAPTA)_in_. The test-pulse trace that follows –80 mV pre-pulse is red. **(C)** Recovery from inactivation. **(D)** Activation (top) and SSI (bottom) curves of Na_V_1.5_WT_ +GFP and Na_V_1.5_A1924T^*_+GFP co-expressing rNa_V_β1/RFP were plotted with the fitted curves of these groups without rNa_V_β1/RFP (presented in **A**, **B**). Current traces recorded in [10 μM Ca^2+^]_in_. The –40 mV trace during activation and the test-pulse that follows the –80 mV pre-pulse during SSI are red. **(E)** Activation (left) and SSI (right) curves with 10 μM Ca^2+^ chelated with HEDTA in the internal solution. **(F)** Activation (left) and SSI (right) curves of Na_V_1.5 variants co-expressing rNa_V_β1/RFP and GFP-Na_V_1.5_K1493del_ were plotted with the fitted curves of these groups without GFP-Na_V_1.5_K1493del_ (presented in **D**). Statistical significance of Va was determined by *t*-test: ns, not significant;^*^p < 0.05; ^∗∗^*p* < 0.01; ^∗∗∗^, *p* < 0.001 (see Table 2).

We wanted to examine whether the non-conducting channel Na_V_1.5_K1493del_ affects the current of the co-expressed Na_V_1.5_A1924T__^*_ variant, consistent with the compound heterozygosity observed in the patient. We tested Ca^2+^-dependent gating properties of I_Na_ in view of previous studies that included K1493 and A1924 residues in structural elements that bind the Ca^2+^-sensor CaM: DIII-IV linker and the CT, respectively. When the I_Na_ conducting variants Na_V_1.5_A1924T^*_ or Na_V_1.5_WT_ were co-expressed with the non-conducting variant Na_V_1.5_K1493del_, the latter was expressed as a GFP-fused channel to attest its co-expression in cells that displayed I_Na_. Co-expression of GFP-Na_V_1.5_K1493del_ resulted in a 3 mV depolarizing shift of the activation curve of Na_V_1.5_A1924T__^*_ in the presence of [10 μM Ca^2+^]_in_ but not in the absence of [Ca^2+^]_in_. Co-expression of GFP-Na_V_1.5_K1493del_ with Na_V_1.5_WT_ resulted in a similar 3.5 mV depolarizing shift of the activation curve, only in [10 μM Ca^2+^]_in_ (Figure 4A and Table 2). Although modest, the depolarizing shift in the activation curve in the presence of Ca^2+^ was significant. The similar current amplitudes (Table 2) and the similar effect in the two unrelated groups argue against the possibility of a recording artifact being the reason for the observed shift.

**TABLE 2:** Gating properties of Na_V_1.5 in different Ca^2+^ chelation.

		10 mM EGTA																				
		**WT + GFP**			**GFP-WT**			A1924T^*^ +GFP			A1924T^*^ +GFP-K1493del			**WT + GFP-K1493del**			**GFP-WT + rβ1**			**GFP-WT + hβ1**		
		**mean**	***n***	**SEM**	**mean**	***n***	**SEM**	**mean**	***n***	**SEM**	**mean**	***n***	**SEM**	**mean**	***n***	**SEM**	**mean**	***n***	**SEM**	**mean**	**n**	**SEM**

activation	Gmax	46.8	18	7.0	47.0	14	6.18	51.7	9	8.9	74.8	7	7.2	38.5	10	6.2	57.94	10	9.25	51.4	12	5.6
	Vrev	42.0	18	2.5	48.8	14	3.0	31.0	9	2.0	35.2	7	3.7	41.6	10	1.7	35.83	10	5.6	34.4	12	4.2
	Va	–36.8	18	1.0	–38.7	14	0.98	–36.8	9	0.9	–36.8	7	1.0	–38.1	10	0.8	–37.8	10	2.5	–39.5	12	2.35
	Ka	6.15	18	0.3	6.23	14	0.3	6.4	9	0.4	5.6	7	0.2	5.5	10	0.4	6.2	10	0.7	5.6	12	0.54

		**10 μM Ca^2+^ (using BAPTA)**												**0 Ca^2+^ (10 mM BAPTA)**											
		**WT + GFP**			**WT + GFP-K1493del**			A1924T^*^ + GFP			A1924T^*^ + GFP-K1493del			**WT + GFP**			**WT + GFP-K1493del**			A1924T^*^ + GFP			A1924T^*^ + GFP-K1493del		
		**mean**	**n**	**SEM**	**mean**	**n**	**SEM**	**mean**	**n**	**SEM**	**mean**	**n**	**SEM**	**mean**	**n**	**SEM**	**mean**	**n**	**SEM**	**mean**	**n**	**SEM**	**mean**	**n**	**SEM**

activation	Gmax	49.5	13	8.1	46.4	15	6.4	47.4	7	9.6	52.8	22	6.0	73.2	7	10.8	45.1	7	8.9	53.2	15	7.7	36.8	16	3.9
	Vrev	42.4	13	1.9	46.2	15	4.2	40.1	7	2.2	45.4	22	2.6	51.0	7	1.2	57.8	7	6.1	54.0	15	2.0	58.1	16	2.1
	Va	–37.6	13	0.6	$-34.1{}^{a}$	15	0.6	–37.8	7	0.7	$-34.8{}^{b,c}$	22	0.5	–32.2	7	1.3	–31.7	7	1.2	–32.6	15	0.7	–31.0	16	0.5
	Ka	6.4	13	0.3	7.2	15	0.3	7.5	7	0.4	7.4	22	0.3	5.9	7	0.4	5.8	7	0.5	7.2	15	0.4	7.4	16	0.4
Amplitude (pA)		−2872	13	502	−2521	15	323	−2583	7	532	−2836	22	308	−4509	7	647	−3002	7	578	−3330	15	513	−2523	16	230
SSI	Va	–83.8	20	0.8	–83.8	5	0.8	–86.0	15	0.7	–85.2	10	0.8	–81.7	29	1.0	$-76.1{}^{d}$	10	1.2	$-86.2{}^{e}$	24	1.2	$-79.8{}^{f}$	22	0.9
	Ka	6.3	20	0.4	7.0	5	0.8	5.7	15	0.2	5.9	10	0.2	5.0	29	0.1	5.3	10	0.2	5.8	24	0.2	5.6	22	0.2
Rec. IA	A	0.87	16	0.02				0.86	15	0.02	0.80	9	0.02	0.88	18	0.02				0.85	18	0.02	0.87	13	0.01
	τ	7.1	16	0.4				7.2	15	0.5	5.7	9	0.2	5.1	18	0.4				5.1	18	0.4	4.4	13	0.4

a, *p* < 0.001 compared to WT+ GFP; b, *p* = 0.009 compared to A1924T^*^+ GFP; c, *p* = 0.003 compared to WT+ GFP; d, *p* = 0.005 compared to WT+ GFP; e, *p* = 0.006 compared to WT+ GFP; f, *p* < 0.001 compared to A1924T^*^+ GFP.																									

		**10 μM Ca^2+^ (using BAPTA)**												**0 Ca^2+^ (10 mM BAPTA)**											
		**WT + rβ1**			**WT + GFP-K1493del + rβ1**			A1924T^*^ + rβ1			A1924T^*^ + GFP-K1493del + rβ1			**WT + rβ1**			**WT + GFP-K1493del + rβ1**			A1924T^*^ + rβ1			A1924T^*^ + GFP-K1493del + rβ1		
		**mean**	**n**	**SEM**	**mean**	**n**	**SEM**	**mean**	**n**	**SEM**	**mean**	**n**	**SEM**	**mean**	**n**	**SEM**	**mean**	**n**	**SEM**	**mean**	**n**	**SEM**	**mean**	**n**	**SEM**

activation	Gmax	39.2	11	4.6	44.7	6	6.8	68.5	6	10.5	75.6	8	11.9	63.9	20	5.13	59.2	9	8.2	38.0	11	6.0	52.5	12	8.0
	Vrev	46.8	11	2.0	51.6	6	4.2	43.0	6	1.0	38.1	8	2.2	50.6	20	1.76	52.2	9	1.8	50.4	11	1.5	52.2	12	2.0
	Va	$-32.6{}^{g}$	11	0.6	–32.6	6	0.5	$-39.9{}^{h}$	6	0.9	–37.9	8	0.9	–31.9	20	0.8	–30.7	9	0.4	–31.7	11	0.7	–32.1	12	0.6
	Ka	6.9	11	0.4	6.4	6	0.6	6.2	6	0.5	6.7	8	0.3	6.1	20	0.3	6.7	9	0.4	7.7	11	0.3	7.5	12	0.5
Amplitude (pA)		−2248	11	290	−2789	6	349	−4270	6	704	−4043	8	670	−3836	20	314	−3548	9	484	−2187	11	356	−3124	12	476
SSI	Va	$-78.0{}^{i}$	12	0.9	–76.6	10	0.7	$-86.3{}^{j}$	6	0.8	–85.2	6	1.3	$-74.5{}^{k,l}$	15	0.7	–74.1	13	0.7	$-83.2{}^{m}$	11	0.9	$-78.5{}^{n}$	15	1.0
	Ka	5.2	12	0.3	5.3	10	0.3	5.0	6	0.3	4.9	6	0.3	4.8	15	0.2	5.6	13	0.2	5.25	11	0.3	4.91	15	0.1

g, *p* < 0.001 compares to WT+ GFP; h, *p* < 0.001 compares to WT+rβ1; i, *p* < 0.001 compares to WT+ GFP; j, *p* < 0.001 compares to WT+rβ1; k, *p* = 0.007 compared to WT+rβ1 in 10 μM Ca^2+^; l, *p* < 0.001 compares to WT+ GFP; m, *p* < 0.001 compares to WT+rβ1; n, *p* = 0.003 compared to A1924T^*^+rβ1.																									

			**10 μM Ca^2+^ (using HEDTA)**																						
			**WT**							**WT + rβ1**						A1924T^*^						A1924T^*^ + rβ1			
								
				**mean**		**n**	**SEM**		**mean**			**n**	**SEM**			**mean**		**n**		**SEM**		**mean**	**n**		**SEM**

activation		Gmax		75.5		10	8.8		46.9			10	8.1			57.2		5		18.4		73.8	5		17.8
		Vrev		45.0		10	2.3		40.7			10	3.2			48.8		5		3.1		44.2	5		1.4
		Va		–37.2		10	1.0		$-33.7{}^{o}$			10	1.2			–37.9		5		0.8		$-38.4{}^{p}$	5		1.4
		Ka		6.3		10	0.2		6.9			10	1.0			5.7		5		0.4		5.8	5		0.6
Amplitude (pA)				−4633		10	507		−2880			10	547			−3764		5		1241		−4622	5		1192
SSI		Va		–85.6		6	1.3		$-80.1{}^{q}$			5	0.9			–84.4		7		1.7		$-85.1{}^{r}$	7		1.1
		Ka		6.0		6	0.4		6.4			5	0.7			5.8		7		0.5		4.8	7		0.1

**o, *p* = 0.037 compared to WT; p, *p* = 0.033 compared to WT+rβ1; q, *p* = 0.007 compared to WT; r, *p* = 0.008 compared to WT+rβ1. SSI, steady-state inactivation; rec IA, recovery from inactivation. Significance between two groups was tested using a two-tailed *t*-test, in the same solution unless otherwise mentioned. β subunits were expressed with RFP in the same vector (β1/RFP).**

As previously reported with Na_V_1.5_A1924T_ (Potet et al., 2009), the SSI curve of Na_V_1.5_A1924T^*_ shifts to hyperpolarized voltages compared to Na_V_1.5_WT_, in the absence, but not in the presence, of [Ca^2+^]_in_. Co-expression of GFP-Na_V_1.5_K1493del_ caused a depolarizing shift of SSI of Na_V_1.5_WT_ and Na_V_1.5_A1924T^*_ by 5.6 and 6.4 mV, respectively, in the absence of [Ca^2+^]_in_, but did not affect SSI properties in the presence of [10 μM Ca^2+^]_in_ (Figure 4B and Table 2). Recovery from inactivation induced by a 1 s depolarizing pulse was not affected by GFP-Na_V_1.5_K1493del_ co-expression (Figure 4C and Table 2).

To conclude, expression of non-conductive GFP-Na_V_1.5_K1493del_ altered the gating properties of co-expressed conducting channels, in a Ca^2+^-dependent manner: a slight decrease in the voltage dependency of activation curve in [10 μM Ca^2+^]_in_, and an increase in Na_V_1.5 availability following inactivation, in the absence of [Ca^2+^]_in_.

Since Na_V_1.5_K1493del_ plasma membrane expression was ∼40% of Na_V_1.5_WT_, and Na_V_β1 enhanced Na_V_1.5_K1493del_ membrane expression (Figure 3C), we decided to examine the Ca^2+^-dependent gating effects of functional channels (Na_V_1.5_WT_ or Na_V_1.5_A1924T^*_) co-expressed with Na_V_β1/RFP and GFP-Na_V_1.5_K1493del_.

Na_V_β1 expression induced a Ca^2+^-independent depolarizing shift in Na_V_1.5_WT_ SSI (Wingo et al., 2004), nevertheless, we observed a Ca^2+^-dependent regulation of the activation. Na_V_β1- expression induced a depolarizing shift in Na_V_1.5_WT_ activation curve, in the presence of [10 μM Ca^2+^]_in_ but not in the absence of [Ca^2+^]_in_ (Table 2 and Figure 4D). A1924T^*^ mutation eliminated Na_V_β1-dependent effects on gating. Co-expression of Na_V_β1 did not shift the activation or SSI curves of Na_V_1.5_A1924T^*_, in either the absence or presence of [Ca^2+^]_in_ (Figures 4D,E and Table 2). The same effect was observed when 10 μM Ca^2+^ were chelated with HEDTA instead of BAPTA (Figure 4E).

Co-expression of GFP-Na_V_1.5_K1493del_ had no significant effect on activation or SSI curves of Na_V_1.5_WT_ +Na_V_β1, in either the absence or presence of [Ca^2+^]_in_. Nevertheless, GFP-Na_V_1.5_K1493del_ co-expression right-shifted the SSI curve of Na_V_1.5_A1924T^*_ + Na_V_β1, only in the absence of [Ca^2+^]_in_ (Figure 4F and Table 2), similar to the effect recorded without Na_V_β1 (Figure 4B). GFP-Na_V_1.5_K1493del_ co-expression did not significantly change the activation properties of Na_V_1.5_A1924T^*_ + Na_V_β1 (Table 2). Thus, the mutation A1924T^*^ blunted Na_V_β1-induced gating regulation and partially restored the effects that were induced by GFP-Na_V_1.5_K1493del_ co-expression.

We suggest that Na_V_β1 directly affects Na_V_1.5 Ca^2+^-dependent gating in addition to its role in expression regulation. Our results demonstrate, for the first time, that Na_V_β1-regulates Na_V_1.5 gating in a Ca^2+^-dependent manner, and that Na_V_β1-induced regulation mechanism includes the A1924 residue. In summary, Na_V_1.5 Ca^2+^-sensitivity involves multiple elements, including both CaM-interacting elements: CT and DIII-IV, and possibly a protein complex that includes more than one Na_V_1.5 channel.

### K1493del Mutation Modulates CaM-Na_V_1.5 Interaction

We wanted to examine CaM-interaction with Na_V_1.5 variants, since CaM directly interacts with Na_V_1.5 at the two domains where variants were found in the proband: A1924T located in the conserved IQ-domain in the cytoplasmic CT (Gabelli et al., 2014; Wang et al., 2014), and K1493del in the proximal segment of the cytoplasmic DIII-IV linker (Potet et al., 2009; Sarhan et al., 2012; Johnson et al., 2018; Figure 1C). We explored the interactions of CaM with Na_V_1.5 expressed in HEK cells by a pull-down essay using CaM-coated agarose beads.

**FIGURE 5:**
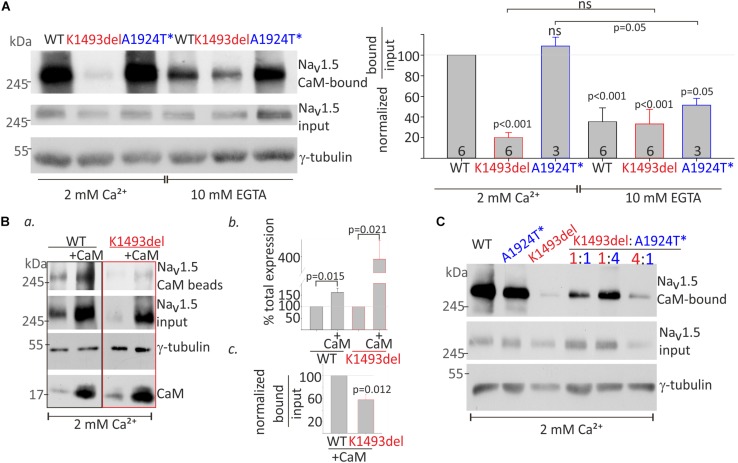
CaM-interaction with Na_V_1.5 variants **(A)**. HEK cells were transfected with Na_V_1.5_WT_, Na_V_1.5_K1493del_, and Na_V_1.5_A1924T^*_ together with rNa_V_β1. Lysates from transfected groups were pulled-down with CaM-agarose beads in Ca^2+^ and EGTA. Left, a representative Western blot from a pull-down assay. Right, the CaM-bound fraction was normalized to input level, and presented as % of Na_V_1.5_WT_ in 2 mM Ca^2+^, in the same experiment. Statistical significance was determined by one-way ANOVA followed by Holm–Sidak test. *P*-value compared to Na_V_1.5_WT_ in 2 mM Ca^2+^ is indicated above each bar. **(B)** Na_V_1.5_WT_ and Na_V_1.5_K1493del_ with or without co-expressed CaM were pulled-down with CaM-agarose beads in 2 mM Ca^2+^. **(a)** CaM- bound Na_V_1.5, and total input levels of Na_V_1.5, CaM and γ-tubulin were detected in Western-blot. **(b)** Input Na_V_1.5+CaM levels were normalized to Na_V_1.5 without CaM, from the same experiment, *N* = 4. **(c)** CaM-bound fractions were normalized to input levels, and are presented as % of Na_V_1.5_WT_+CaM in the same experiment, *N* = 3. Statistical significance was estimated by *t*-test. **(C)** HEK cells transfected with equal total Na_V_1.5 α-subunit (7.5 μg; ratio 1:1, 3.75 μg of each construct; ratio 1:4, 1.5, and 6 μg of the constructs indicated) together with rNa_V_β1. Lysates from transfected groups were pulled-down with CaM-agarose beads in 2 mM Ca^2+^. This is a representative experiment out of two.

CaM interaction in the presence of 2 mM Ca^2+^ (Ca^2+^/CaM interaction) and in the absence of Ca^2+^ (apo-CaM interaction in 10 mM EGTA) with Na_V_1.5_WT_ and mutants in the same experiment, were normalized to Ca^2+^/CaM-Na_V_1.5_WT_ interaction. Co-expression of Na_V_β1 enabled comparable protein expression levels of Na_V_1.5_K1493del_ and Na_V_1.5_WT_. A three-fold reduction in CaM-Na_V_1.5_WT_ interaction in the absence of Ca^2+^ (from 100% with Ca^2+^ to 35 ± 13% without Ca^2+^), and a two-fold reduction in CaM-Na_V_1.5_A1924T^*_ interaction in the absence of Ca^2+^ (from 108 ± 8% with Ca^2+^ to 51 ± 6% without Ca^2+^) were observed (Figure 5A). Ca^2+^/CaM interaction with Na_V_1.5_K1493del_ were strongly reduced (20 ± 5% of Na_V_1.5_WT_). The Ca^2+^/CaM and apo-CaM interaction with Na_V_1.5_K1493del_ were not significantly different, indicating an impaired Ca^2+^-dependent interaction (Figure 5A).

CaM co-expression enhanced total-expression of Na_V_1.5_WT_ by 161 ± 18% and Na_V_1.5_K1493del_ by 387 ± 93% (Figures 5Ba,b), suggesting that despite a reduction in Ca^2+^/CaM interaction, CaM regulates the expression levels of Na_V_1.5_K1493del_. Upregulation of Na_V_1.5_K1493del_ expression in the presence of over-expressed CaM enabled quantification of CaM-Na_V_1.5_K1493del_ interaction. The ratio of Na_V_1.5 bound-to-input levels in 2 mM Ca^2+^ was lower in Na_V_1.5_K1493del_ compared to Na_V_1.5_WT_ (58 ± 10%, Figures 5Ba,c). Thus, CaM over-expression was not able to fully restore the reduction in Ca^2+^/CaM-Na_V_1.5_K1493del_ interaction.

We wanted to determine whether the combination of the two mutated channels, as presented in the patient, affects the overall binding of the expressed channels to CaM. We hypothesized that a cross-talk between Na_V_1.5 proteins would result in cooperative CaM-interaction and that reduced Ca^2+^/CaM-interaction in Na_V_1.5_K1493del_ would interfere with overall Ca^2+^/CaM-interaction. Our results did not support this hypothesis. The extent of overall channel binding to CaM beads in 2 mM Ca^2+^ varied proportionally to the ratio of expressed Na_V_1.5_K1493del_ and Na_V_1.5_A1924T^*_: the more Na_V_1.5_K1493del_ – the less overall CaM binding (Figure 5C).

### The Mechanism of Na_V_1.5_K1493del_ Loss-of-Function

**FIGURE 6:**
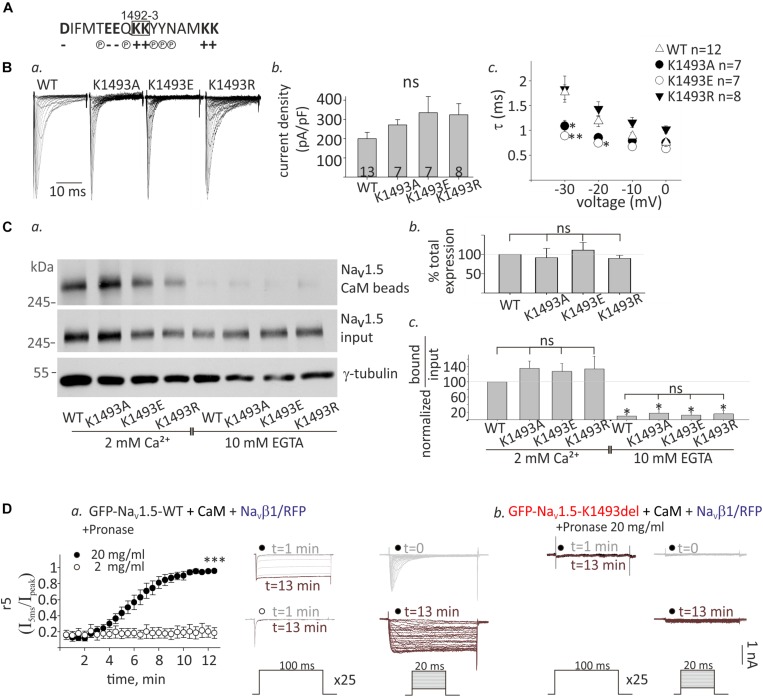
Functional and biochemical role of K1493 residue. **(A)** The amino acid sequence of residues 1484–1500 in Na_V_1.5. The double lysines 1492-3 are marked in a square. Amino acid’s charge (+ or–) or polarity (P) are indicated. **(B)** Na_V_1.5 variants co-expressed with rNa_V_β1. **(a)** Representative I_Na_ traces recorded in (10 mM EGTA)_in_. **(b)** Maximal current densities. **(c)** Time constant (τ) obtained by fitting a single exponential to the decay phase of the currents in (10 mM EGTA)_in_ at four voltages. Statistical significance was determined by one-way ANOVA followed by Holm–Sidak test (compared to Na_V_1.5_WT_). **(C)** Cells were transfected with Na_V_1.5_K1493A/E/R_ together with rNa_V_β1. **(a)** Example of a pull-down experiment with CaM-agarose beads, in Ca^2+^ and EGTA. **(b)** The total expression as quantified in EGTA, and normalized to Na_V_1.5_WT_ (*N* = 3). **(c)** Pull-down experiments with CaM-agarose beads. The CaM-bound fraction was normalized to input level and quantified as % of Na_V_1.5_WT_ in 2 mM Ca^2+^ (*N* = 3). Statistical significance was determined by one-way ANOVA, followed by Holm–Sidak test, compared to WT in 2 mM Ca^2+^. ^∗∗^*p* < 0.01; ^*^p < 0.05. **(D)** GFP-Na_V_1.5_WT_ **(a)** or GFP-Na_V_1.5_K1493del_ **(b)** coexpressed with rNa_V_β1/RFP and CaM in HEK cells. 2 or 20 mg/ml pronase were added to (10 mM EGTA)_in_ pipette solution. *t* indicates the time from the beginning of the recording. **(a)** Currents elicited from –120 mV to –20 mV for 100 ms, every 30 s. Between pulses, the cells were held at –120 mV. Inactivation was evaluated by r_5_, which is the fraction of current that remained after 5 ms at –20 mV (I_5ms_/I_peak_), *n* = 5 in each group (left). Representative currents during repetitive steps to –20 mV (middle), and activation protocols in the same cell, at different time points (right). **(b)** Recording from cells expressing GFP-Na_V_1.5_K1493del_ with 20 mg/ml pronase in the pipette solution. Currents during repetitive depolarization (left) and activation protocols (right) at different time points are presented. This is a representative cell out of 12 cells.

The lysine doublet in position 1492-3 is positively charged and is located in a region rich with polar and charged residues that could potentially contribute to protein-protein interactions (Figure 6A). These residues are located in a conserved helical structure, downstream of the IFM motif that confers fast inactivation (West et al., 1992). We hypothesized that a salt-bridge could affect channel function and CaM interaction. To test this, we created three Na_V_1.5 mutants, where the lysine in position 1493 was mutated to the neutral residue alanine, Na_V_1.5_K1493A_, the negative residue glutamate, Na_V_1.5_K1493E_, or another positive residue, arginine, Na_V_1.5_K1493R_.

Analysis of Na_V_1.5 currents revealed that all three mutated channels were functional (Figures 6Ba,b). However, inactivation properties were altered. Using a single-exponential fit to estimate the inactivation time constant, τ, we found that when the positive charge in residue 1493 was conserved (K1493R), inactivation rate was similar to WT, as previously reported (Li et al., 2009). However, the inactivation rate was increased when K1493 was changed to an uncharged or a negatively charged residue (K1493A/E, respectively, Figure 6Bc). These results indicate that the electric charge in position 1493 is important for fast-inactivation kinetic properties, but not for the total function.

Expression analysis showed that the total protein levels of the three mutants Na_V_1.5_K1493A/E/R_ were not significantly different from Na_V_1.5_WT_ (Figures 6Ca,b). CaM-interaction with Na_V_1.5_K1493A/E/R_ was Ca^2+^-dependent, similar to Na_V_1.5_WT_ (Figures 6Ca,c). This set of experiments demonstrates that, unlike the robust effect of the K1493 deletion mutation, the biochemical and functional characteristics of Na_V_1.5 are not highly dependent on the electric charge in K1493 residue.

We further explored the mechanism of Na_V_1.5_K1493del_ loss-of-function in view of the mutation location, in Na_V_1.5 inactivation gate. We hypothesized that Na_V_1.5_K1493del_ is constitutively inactivated resulting in a channel-pore block. To address this hypothesis, we used the protease pronase that selectively destroys the inactivation of sodium channels while leaving activation intact (Armstrong et al., 1973). HEK cells were transfected with GFP-Na_V_1.5_WT_ or GFP-Na_V_1.5_K1493del_ together with both CaM and Na_V_β1/RFP to allow maximal expression of Na_V_1.5 variants. Dialysis of 20 mg/ml pronase into the cell through the patch pipette gradually relieved the inactivation of Na_V_1.5_WT_ I_Na_ until its complete elimination after 12 min. The effect on I_Na_ inactivation was dose-dependent and 2 mg/ml pronase did not eliminate the inactivation in 12 min (Figure 6Da). Cells expressing GFP-Na_V_1.5_K1493del_ and Na_V_β1/RFP in addition to CaM had no I_Na_. Dialysis of 20 mg/ml pronase, in the same protocol used for Na_V_1.5_WT_, did not restore Na_V_1.5_K1493del_ current. We conclude that the reasons for Na_V_1.5_K1493del_ loss-of-function are beyond a change in the inactivation properties, and probably involve structural and additional functional perturbations.

## Discussion

Sinus-bradycardia and cardiac conduction-disease in the proband were associated with novel heterozygous *SCN5A* variants composition, K1493del in DIII-IV linker and A1924T^*^ in the CT of Na_V_1.5. Deletion of K1493 caused a complete loss of Na_V_1.5 function. Surprisingly, the expression of the non-conducting Na_V_1.5_K1493del_ affected Ca^2+^-dependent gating properties of co-expressed conducting channels. Moreover, Ca^2+^-dependent CaM-Na_V_1.5 interaction was impaired in Na_V_1.5_K1493del_. A Ca^2+^-dependent Na_V_β1 modulation that was characterized in Na_V_1.5_WT_ currents, was impaired in the Na_V_1.5_A1924T^*_ variant. These results highlight the significance of Na_V_1.5 DIII-IV linker in channel function and CaM-interaction and suggest that the Ca^2+^-sensing machinery of Na_V_1.5 involves Na_V_β1 and more than one monomeric Na_V_1.5 channel.

### Function and Biogenesis of Na_V_1.5_K1493del_

Sodium channelopathies usually occur due to disruption of two mechanisms: gating and/or biogenesis, a process that includes synthesis, folding, assembly of the macromolecular complex, trafficking to the plasma membrane and localization in cell surface compartments (Chen-Izu et al., 2015). Co-expression of the auxiliary proteins Na_V_β1 and CaM increased Na_V_1.5_WT_ and Na_V_1.5_K1493del_ total expression (Figures 3B, 5Bb), possibly by serving as chaperons, suggesting a proper biogenesis regulation of Na_V_1.5_K1493del_ by these proteins. The Na_V_β1 and the ubiquitous CaM are expressed throughout the heart and the cardiac conduction system (O’malley and Isom, 2015). Thus, we assume that expression levels of Na_V_1.5_K1493del_ in the human heart are close to the physiological levels of Na_V_1.5_WT_.

The non-conducting channel Na_V_1.5_K1493del_ did not exert a dominant-negative effect on the macroscopic current density of Na_V_1.5_WT_ or Na_V_1.5_A1924T^*_ in HEK cells, or on endogenous I_Na_ of HL-1 atrial cells. Hence, Na_V_1.5_K1493del_ does not impair biogenesis of other sodium channels, a mechanism reported in other Na_V_1.5 mutants (Keller et al., 2005; Clatot et al., 2012; Hoshi et al., 2014).

Deletion of K1493 resulted in a loss of I_Na_. Changing K1493 electrostatic properties did not reduce I_Na_ but only modified inactivation kinetics (Figure 6B). Based on the location of the mutation in the inactivation gate, a constitutive inactivation state could account for Na_V_1.5_K1493del_ loss-of-function. However, pronase, a protease that relieves the inactivation of sodium channels following the digestion of DIII-IV linker (Stühmer et al., 1989), did not restore Na_V_1.5_K1493del_ currents (Figure 6D). Possible explanations are, first, that K1493del mutation altered Na_V_1.5 DIII-IV linker conformation to hinder the access of pronase to its target sequence. In an inactivated state, the IFM motif acts as a latch of a hinged-lid that docks within the pore. If K1493del does not allow a release of the latch in resting potentials, then the channel remains locked in an inactivated state, and the substrate of pronase might not be exposed to the cytoplasm. A second possible explanation is that K1493 deletion not only destabilized the inactivation gate but also lead to a major deformation that obstructed the pore’s cytosolic mouth. In this case, a twisted DIII-IV linker conformation results in a global interference in the protein structure and distal interaction with other Na_V_1.5 cytosolic elements. Both options imply that the lysines in position 1492-3 are pivotal residues in Na_V_1.5 gate and that removal of one lysine alters fundamental functional and structural elements in the channel gate.

Unlike a previous report (Zumhagen et al., 2013), we were unable to record I_Na_ in HEK cells expressing Na_V_1.5_K1493del_. In the previous and the current reports, Na_V_1.5_K1493del_ was expressed in HEK cells, and the patch-clamp solutions and experimental conditions were essentially similar. We tested several mechanisms that may have contributed to the discrepancy: (1) Impaired cellular expression: total and surface expression of Na_V_1.5_K1493del_ was confirmed by Western blot and biotinylation assay (Figure 3). (2) Low transfection of Na_V_1.5 α-subunit: currents were measured in cells expressing GFP-labeled α-subunit (Na_V_1.5_WT_ and Na_V_1.5_K1493del_), and the amount of DNA used for transfection of Na_V_1.5_K1493del_ was three-fold higher. (3) Functional variability between Na_V_β1 species: Na_V_β1 subunit from two species were used (rat and human; the human Na_V_β1 was previously used). (4) Low expression of Na_V_β1 subunit: Na_V_β1 was co-expressed with a fluorescent marker in a bicistronic vector. (5) Finally, the coding sequences of the constructs were fully sequenced, and two separately constructed α-subunit mutants (Na_V_1.5_K1493del_ and GFP-Na_V_1.5_K1493del_) were tested. In summary, our results show that Na_V_1.5_K1493del_ is a loss-of-function mutation due to a gating rather than a biogenesis defect, and the reason for the discrepancy with the previous report could not be determined.

### DIII-IV Linker Mediates Ca^2+^-Dependent Na_V_1.5-CaM Interaction

The Ca^2+^-sensing machinery of Na_V_1.5 may include the DIII-IV linker, CT and the Ca^2+^ sensor CaM. Interaction of CaM with DIII-IV linker was studied mainly using small peptides (Kim et al., 2004; Potet et al., 2009; Sarhan et al., 2012; Yan H. et al., 2017; Johnson et al., 2018), but the relevance of DIII-IV linker to the overall CaM-binding complex in the full Na_V_1.5 protein is unclear. The binding affinities of Na_V_1.5-CT peptide to CaM were not Ca^2+^-sensitive (Wang et al., 2014), while Ca^2+^-dependent enhancement in CaM binding was measured with DIII-IV linker segments (Sarhan et al., 2009; Sarhan et al., 2012; Johnson et al., 2018). We detected a novel property of CaM interaction with full Na_V_1.5 expressed in cells: Ca^2+^/CaM was stronger than apo-CaM interaction, in Na_V_1.5_WT_ and the variants Na_V_1.5_A1924T^*_ (Figure 5A) and Na_V_1.5_K1493A/E/R_ (Figure 6C) when using pull-down assay. K1493del blunted the Ca^2+^-dependent CaM-interaction: Ca^2+^/CaM-Na_V_1.5_K1493del_ interaction was similar to apo-CaM-interaction with Na_V_1.5_WT_ or Na_V_1.5_K1493del_. These results highlight the role of DIII-IV linker in CaM binding complex, suggesting that the Ca^2+^-dependent CaM-interaction with Na_V_1.5 DIII-IV linker contributes, directly or indirectly, to the overall enhancement in Ca^2+^/CaM-Na_V_1.5 interaction.

### The Na_V_1.5 Mutation A1924T Does Not Eliminate Ca^2+^-Dependent CaM-Interaction

A1924T mutation reduced Ca^2+^/CaM-interaction in a CT peptide (Wang et al., 2014). However, the length of Na_V_1.5 CaM-interacting segments was shown to be crucial for determining binding affinities to CaM [(Wang et al., 2014; Johnson et al., 2018) vs. (Sarhan et al., 2012)]. When we pulled-down the full-length expressed Na_V_1.5, the interaction between Ca^2+^/CaM and Na_V_1.5_A1924T^*_ was not weakened compared to Na_V_1.5_WT_, unlike the reports in CT segments. However, in the absence of Ca^2+^, Na_V_1.5_A1924T^*_-CaM interaction was slightly higher compared to Na_V_1.5_WT_, so the “net” Ca^2+^-dependent change in CaM interaction was diminished. Our results point that the binding of CaM to a native Na_V_1.5 channel is probably determined by multiple segments, e.g., both the CT and the DIII-IV linker. CaM affinities to separate segments may not reflect the full dynamic interaction. Understanding the mode of integration of all Na_V_1.5-CaM binding elements within the complete channel protein is indispensable for resolving the mechanism of CaM-regulation in a physiological context. Pioneering cryogenic electron microscopy (cryo-EM) studies were able to capture the cytosolic complex of sodium channels (Shen et al., 2017; Yan Z. et al., 2017), and together with functional and molecular information the understanding of the complex and dynamic Ca^2+^-dependent regulation will be refined.

### Na_V_1.5 Gating Is Ca^2+^-Dependent

Several studies demonstrated that Na_V_1.5 is regulated by Ca^2+^ (Deschenes et al., 2002; Tan et al., 2002; Kim et al., 2004; Wingo et al., 2004; Biswas et al., 2009; Glynn et al., 2015; Gabelli et al., 2016; Abdelsayed et al., 2017), but the molecular details and the physiological relevance remains highly controversial [e.g. (Ben-Johny et al., 2014)]. We showed that Na_V_1.5_K1493del_ is located in the plasma membrane as a non-conducting channel. If individual Na_V_1.5 monomers gate independently of others, the “silent” mutant Na_V_1.5_K1493del_ would not have contributed to overall macroscopic current properties when expressed with other channels in the same cell. Strikingly, expression of the non-conducting mutant channel affected the Ca^2+^-dependent gating of macroscopic current arising from co-expressed conducting channels. Co-expression of Na_V_1.5_K1493del_ similarly altered the Ca^2+^-dependent gating properties of both Na_V_1.5_WT_ and Na_V_1.5_A1924T^*_: a depolarization shift in the voltage-dependent activation with [Ca^2+^]_in_ and SSI-curve in the absence of [Ca^2+^]_in_.

Previous studies provided evidence that Na_V_1.5 proteins are in physical proximity when expressed in cells (Clatot et al., 2012; Mercier et al., 2012; Clatot et al., 2018). This interaction is indirect, via 14-3-3 protein (Clatot et al., 2017). Functional cooperation of the gate has been demonstrated when loss-of-function Na_V_1.5 mutant impaired Na_V_1.5_WT_ gating by a dominant-negative mechanism (Clatot et al., 2018) while several other mutations showed a dominant-negative effect via defective biogenesis that suggests co-trafficking of several channels [reviewed in (Sottas and Abriel, 2016)]. Here, the expression of Na_V_1.5_K1493del_ did not cause a dominant-negative loss-of-function effect (Figures 2B,C). We propose that the loss of Ca^2+^/CaM-interaction due to K1493del mutation prompted changes in the Ca^2+^-dependent gating of co-expressed conducting channels. Thus, our results support a cooperative gating regulation of multimeric-Na_V_1.5 complex and suggest that this mechanism involves a Ca^2+^/CaM-regulated component. To note, CaM plays a critical role in the functional coupling of the structurally homologous calcium channel, Ca_V_1.2 (Dixon et al., 2015). In summary, the mutation K1493del underlines the role of DIII-IV linker in Ca^2+^-dependent regulation of coupled Na_V_1.5 channels. The involvement of CaM and the details of Ca^2+^-dependent regulation of Na_V_1.5’s cooperative gating mechanism are yet to be determined.

### Na_V_β1 Regulates Na_V_1.5 Gating Through CaM Interacting Domains

The interaction between monomeric Na_V_1.5 during channel biogenesis is mediated by Na_V_β1 (Mercier et al., 2012), but the involvement of Na_V_β1 in the gating of coupled channels or in Ca^2+^-dependent mechanisms have not been elucidated. The reported effects of Na_V_β1 on Na_V_1.5 gating parameters in mammalian expression systems are conflicting, generally suggesting that the cellular environment is critical for channel function (Calhoun and Isom, 2014). Similar to previous reports (An et al., 1998; Wingo et al., 2004; Zhu et al., 2017) we showed that Na_V_β1 co-expression right-shifted Na_V_1.5_WT_ SSI curve (Figure 4D, bottom). Further, we showed that Na_V_β1-induced a right-shift in Na_V_1.5_WT_ activation curve in the presence, but not in the absence, of Ca^2+^ (Figure 4D, top). This provides an indication for the role of Ca^2+^ in Na_V_β1 regulation.

GFP-Na_V_1.5_K1493del_ altered Na_V_1.5_WT_ gating properties only when Na_V_β1 was not expressed, whereas in the presence of Na_V_β1, GFP-Na_V_1.5_K1493del_ expression did not affect gating. Na_V_β1 was shown to modulate the voltage-sensor of Na_V_1.5 domain IV (DIV, Figure 1C), and is believed to localize in close proximity to DIII-IV linker (Yan Z. et al., 2017; Zhu et al., 2017). Thus, we propose that Na_V_β1 is intricately involved in the Ca^2+^-dependent gating regulation, and can modify the contribution of DIII-IV linker to Na_V_1.5 channels’ cooperative-gating modulation.

Both effects of Na_V_β1 on Na_V_1.5_WT_ were eliminated in Na_V_1.5_A1924T^*_: the gating modulations and the loss of Na_V_1.5_K1493del_ effect, indicating that A1924 residue may mediate Na_V_β1 regulation. Interestingly, structural dimers were found in the CT of Na_V_1.5. In the dimer, position 1924 is located in the interface of Na_V_1.5 CT dimer, and A1924T mutation was shown to attenuate these interactions (Gabelli et al., 2014). In view of the structural and functional data, we suggest that cytosolic interaction between Na_V_β1 and the IQ domain(s) in Na_V_1.5 cytosolic CT, including A1924 residue, are involved in the regulation of Na_V_1.5-gate. Since both DIII-IV linker and A1924T are thought to be included in the CaM-Na_V_1.5 interaction complex, we postulate that Na_V_β1 is part of a cytosolic CaM-interaction complex and a dynamic modulator of a complexed Ca^2+^-regulated gate that comprises DIII-IV linker and the CT of multiple Na_V_1.5 channels.

### The Effect of Na_V_1.5 Mutants on the Clinical Properties

Compound heterozygosity in *SCN5A* was associated with increased arrhythmic expression compared to heterozygotes in the same family. The clinical phenotype included severe bradycardia and conduction disease, which represents a reduction in electric activity of both the sinus-atria and the conduction system. Among the two mutations that were detected in the proband, the most striking biophysical feature is the loss-of-function due to K1493del mutation, which would lead to a 50% reduction in I_Na_ and haploinsufficiency. The changes in gating that arise from the second Na_V_1.5 variant, A1924T^*^, are expected to have a relatively mild impact on cardiac rhythm but might have added to the pathological expression on the background of Na_V_1.5_K1493del_.

The physiological implication of Ca^2+^ modulation of Na_V_1.5 is heart-rate dependent. During low-to-normal heart rate, I_Na_ transient of the cardiac action-potential precedes the Ca^2+^ transient and senses low Ca^2+^ levels. Repetitive and fast Ca^2+^ transients, during tachycardia, are expected to reveal Ca^2+^-dependent conformational changes in protein complex formation that are limited by the association/dissociation rate, like Na_V_1.5-CaM direct interaction, and other distal Ca^2+^-induced pathways.

Na_V_1.5 channels are physiologically modulated by Na_V_β1 in cardiac cells (Edokobi and Isom, 2018). Interestingly, A1924T^*^ mutant blunted Na_V_β1- induced gating modulation. Close to resting potential and in the absence of Ca^2+^, the availability of Na_V_1.5_A1924T^*_ + Na_V_β1 channels to open is two times lower than that of Na_V_1.5_WT_ + Na_V_β1, which is expected to reduce the action-potential upstroke during low-to-normal heart rate (Figure 4D), and may explain the BrS phenotype reported in A1924T carriers (Rook et al., 1999). The addition of Na_V_1.5_K1493del_ on top of Na_V_1.5_A1924T^*_ + Na_V_β1 increased the number of Na_V_1.5 channels available to open at rest (Figure 4F, right). This restoration of SSI properties may have moderated the development of BrS ECG pattern in the compound-heterozygote in our study, although a low penetration of A1924T phenotype cannot be ruled out.

In high Ca^2+^, the window current of Na_V_1.5_A1924T^*_ + Na_V_β1 is shifted to hyperpolarized voltages comparing to Na_V_1.5_WT_, resulting in increased Na_V_1.5 excitability. We speculate that this property can facilitate conduction and increase the propensity for development of exercise-induced atrial flutter with rapid ventricular response, on the background of bradycardia and conduction disease, that was demonstrated in the compound heterozygote in this study (Figure 1A, right).

It is noteworthy that certain loss-of-function mutations are expressed in BrS phenotype, e.g., A1924T, while others are associated with slow cardiac conduction and bradycardia, without BrS ECG pattern, e.g., K1493del [see also (Hu et al., 2010; Park et al., 2015)]. Possibly, the impaired mechanism that results in a loss-of-function plays a critical role in the downstream expression of the syndrome.

Extrapolation of the biophysical properties of mutated channels to disease expression, in the presented proband, is limited since we cannot determine the relative expression of each allele in the compound heterozygote cardiac cells, and thus we cannot determine the relative functional contribution of each mutated channel. In addition, the role of the polymorphism V1251M was not studied. The use of induced pluripotent stem-cells derived cardiomyocytes (iPSC-CMs), originated from the proband’s cells, would be an attractive approach to investigate how the composite of mutations with distinct biophysical properties results in the cardiac phenotype, in a complex molecular environment, and in dynamic (Ca^2+^) cycles.

## Conclusion

We show that K1493del mutation induces a complete loss-of-function of Na_V_1.5 due to gating, rather than biogenesis, defect. The effect of K1493 deletion is independent of the electric charge in this position. K1493del is associated with impaired CaM-interaction and Ca^2+^-dependent modulation of sodium currents. Na_V_β1 gating regulation is Ca^2+^-dependent and involves the Na_V_1.5 CT. These results highlight the role of Na_V_1.5 DIII-IV linker in Na_V_1.5-CaM-interaction and support a coupling between Na_V_1.5 channels that is implicated in a Ca^2+^-dependent gating mechanism.

## Ethics Statement

The proband and his parents gave written informed consents for both the clinical and genetic studies, which were approved by the Institutional Ethics-Committee of the Sheba Medical Center, Tel-Hashomer (approval 2853/03).

## Author Contributions

SO and EN designed the research. SO planned the experiments, performed patch-clamp experiments, interpreted the data, and wrote the manuscript. EN, RB, DL, and MG clinically evaluated the patient. MG acquired the financial support for the project. LV performed the biochemical experiments. EM prepared the DNA constructs, interpreted the data, and edited the manuscript. CA, JC, and EB performed and analyzed the genetic screen and edited the manuscript.

## Conflict of Interest Statement

The authors declare that the research was conducted in the absence of any commercial or financial relationships that could be construed as a potential conflict of interest.

## Footnotes

1 https://web.stanford.edu/~cpatton/webmaxcE.htm
